# Effects of Educational Attainment and Housing Condition on Self-Rated Health in Old Age: Heterogeneity and Tendency in China

**DOI:** 10.3389/fpubh.2021.774364

**Published:** 2022-01-06

**Authors:** Yuan Yao, Shun Zhang, Aihong Li

**Affiliations:** ^1^Department of Sociology, Hohai University, Nanjing, China; ^2^Department of Sociology, Xi'an Jiaotong University, Xi'an, China; ^3^Department of Sociology, University of Cambridge, Cambridge, United Kingdom

**Keywords:** Chinese elders, self-rated health, educational attainment, housing condition, age pattern

## Abstract

In China, the health of the elderly has long been discussed, but few have investigated the diversity of the aging pattern in later life of this population. Although a large body of literature has approved the positive association between socioeconomic status (SES) and health, it still remains controversial regarding whether the association becomes convergent or divergent in old ages. Using data from China's 2010 and 2015 Inter-census Survey (1‰ sample), this paper explored the role of two key SES indicators, educational attainment and housing condition in modifying the self-rated health of Chinese elders aged 60 and above. We observed the diversified patterns of how educational attainment and housing condition have made an impact on the health of these elders in their old age and the temporal changes of the two SES indicators. We found higher educational attainment and better housing condition can lead to higher self-rated health. This positive significance however diminished with age over time, as we observed from 2010 to 2015, indicating the convergent effects of SES on health in old age. We also found that although educational attainment and housing condition were both positively correlated with health, their effects were differentiated. The influence of educational attainment on health waxed, whereas on housing conditions waned over time. These findings suggested the heterogeneity of health and SES effects among Chinese elders.

## Introduction

Since the 21st century, the population of China is aging because of the increased life expectancy and low fertility. Life expectancy has increased significantly to 76.34 years in 2015 (1), which was about 43 years when the People's Republic of China (PRC) was established in 1949. By the seventh census of the PRC in 2020, there have been 264 million elderly people aged 60 and over in China, accounting for 18.7% of the total population. Such a large elderly population while not enough welfare support urges more research to fulfill the promise of healthy aging under the grand social context of epidemiologic transition in China (2). Meanwhile, chronic diseases have replaced infectious diseases and become the main disease threatening the elderly Chinese. Under this background, the number of literature reviews on social causes of the elderly's health in China has significantly increased, but few have investigated the heterogeneity and tendency of educational attainment and housing condition on the elderly's health.

Previous studies have identified the significant relationship between socioeconomic status (SES) and health. Factors that lead to social stratification, such as occupation, education, income, and race, are highly correlated with health, and SES has been confirmed as the fundamental factor of health inequality (3–7). Life course theory has provided more insights to understand how SES affects health by pointing out that the dynamic pattern between SES and health at the different stages of an individual's life (8, 9). However, disagreements about health differentiation by age groups arise, leading to further theoretical and empirical investigations. It is noticeable that how health differentiates among different SES groups in later life is the core of the debate. Thus, this paper focused on the health of the elderly to examine the elderly's SES disparities in age-related patterns of health.

Using the Inter-census Survey of two waves, this study focused on two key SES indicators, educational attainment, and housing condition, as the measure of SES, to examine the dynamic effects of SES on health. This study contributes to the understanding of how health production responds to rapid economic development and inequality.

## Literature Review

Literature on the health of older adults has proliferated in the past decades. Although many studies consider the relationship between various social factors and the health of older adults, SES–health gradient is the most noticeable. The phenomenon of SES–health gradient has been discussed for a long time (10), since the publication of the Black Report in 1980 (11). To describe the relationship between SES and health, Michael Marmot coined a conception, namely *status syndrome* (12). Status syndrome pointed out that the differentials of health were not just between the poor and non-poor; once the SES increased, the health level would accordingly increase. This is consistent with the concept of *health gradient*, which has been used in many studies that confirmed the robust relationship between SES and health. Due to *flexible resources*, theory of fundamental cause brought more discussion and research about SES and health (13). So far, the bulk of the studies found that SES is a remarkably consistent predictor of health (8, 14–18), and it is not an exception among older adults. Elderly people who have higher SES, score higher on multiple health indicators, such as mortality, self-rated health, activities of daily living (ADL) disability, functional limitations, metabolic syndrome, quality of life, and longevity (19–26). Meanwhile, some scholars have pointed out, it is necessary to pay more attention to the accumulative effects in gerontology (22). Although the importance of SES on health has been widely acknowledged, whether this importance increases or decreases when people get older remains unclear. Both the divergence and convergence effects of SES on health with age have been suggested by life course studies (27–32).

According to the theory of cumulative (dis)advantages, the divergence effect hypothesis holds that the impact of SES factors will expand in the course of an individual's old age (30–32). However, the theory of cumulative advantages proposed by Merton interpreted as the “Matthew effect” in scientific careers, illustrated that “The rich tend to get richer and the potent become more powerful” (33). The theory of cumulative advantages was first used to explore the cumulative advantage in health by Ross and Wu (28). One research reported that advantaged individuals live longer than their less advantaged counterparts, and this inequality will soar throughout their whole life (34). Another research focused on the working-age population has found that the Gini coefficient, which is used to capture the general health inequality, has significantly increased in old age (31). A study based on panel data has also reported the age-varying relationships between SES, health risks, and chronic disease (35). Findings from country to country have also provided some valid evidence for the accumulation hypothesis (31). People with higher SES have a higher level of awareness of health and are more likely to invest in health (36). The gap of a vast of health gradients, measured by self-reported health, physical functioning, and physical well-being, has been found to increase with age (28), regardless of educational attainment or external risks that could lead to SES inequalities and larger health gap over the life course (30).

On the contrary, the convergence effect hypothesis holds the opposite view that the influence of SES factors on individuals weakens when people become old (37). Researchers found that there were no significant disparities with respect to some health indicators among the aged groups, despite the fact that they had very different adulthood (38). It is possible that the influence of biological factors becomes increasingly dominating in the later life stages (39), whereas the importance of SES factors in shaping health declines over the life course. Another study reported that the converging health inequality cannot be explained by selection bias because mortality and health gradients by SES do not significantly vary by age (29). The convergence in health inequalities in late life is also supported by the age-as-leveler hypothesis (40). However, some relevant studies found that differentials on self-rated health, chronic diseases, and physical function, which are the typical measures of health inequality, would decrease or even disappear in old age (41). Together, these research findings support the convergence effect hypothesis.

Yet, there is no agreement on whether it is convergence or divergence of SES on health by the age in the Chinese context. Using Longitudinal data from China Nutrition and Health Survey (CHNS), Chen and Yang found that the health inequality due to educational differentials was cumulative (2). The research of the old population reported that although the prevalence of mortality significantly increases with age, the effects from SES on mortality become significantly larger while divergent by age among the elders aged 80 and above (42). But several studies in China also have found no significant effect of SES on health among elderly adults when compared with younger adults. In addition, another empirical study reported that the continuing SES differentials on ADL as the health indicator support the convergence effect hypothesis (43). Based on the same dataset, other researchers found the divergence effect on some health indicators and the convergence effect on some other health indicators (44).

From the discussion of current literature, we found that the controversy of divergence effect hypothesis and convergence effect hypothesis still exists and co-exists in the study of SES and health (36), essentially in the studies of elders' later life. Why previous empirical studies are mixed? One explanation is the limitation of many sample surveys, which have not included a large number of elders. Another plausible explanation is due to the use of inconsistent health indicators. Thus, to disentangle the two effects, we used data from a nationally representative survey covering the old population and focused on one highly acknowledged measure, self-rated health to investigate the potential heterogeneity of Chinese elders' health and the pattern of SES effect by age (45).

As noted above, there are quite a few indicators to measure SES, including occupation, income, and so on; nevertheless, educational attainment has always stood out. How does education influence health? The first pathway is the allocation function of education; through the process of selecting, sorting, training, and finally allocating, an individual's social positions and social roles are decided (46). It is known that educational attainment is one of the essential indicators of individuals' occupational attainment, including job status and salary from worldwide. The second pathway is the socialization function of education. The process of education internalizes beneficial lifestyles, habits, and attitudes to health (47, 48), through which education helps to cope with stress and other health risks. Lastly, educational attainment makes an impact on health through mediating social psychological resources. Research has found that highly educated individuals are more likely to feel things are under their control (49), which leads to less mental issues (50). Given the important role of education in health, the association between the age-specific rate of health and education is gaining more attention (51). Therefore, in our research, we also treated educational attainment as one of the essential factors that affect elders' health.

Housing plays a critical role in the health of Chinese people. Housing inequality has become one of the key factors that contribute to the increasing socioeconomic disparity of Chinese society (52, 53). The first research on housing inequality in China was conducted in the 1990s when the housing allocation was still centrally planned by the Chinese government (52), which was found to favor people from higher SES backgrounds or with political positions. In the late 1990s, housing inequality between different SES groups was aggravated due to the economic reforms and marketization (54–56). Two decades after the economic reform, housing has become one of the key indicators of an individual's social class (57), and housing has been acknowledged as one of the crucial social determinants of public health (58, 59). The relationship between precarious housing conditions and poor health has been confirmed by both quantitative and qualitative studies (60, 61). One empirical research on health in late life reported that housing has overridden income in determining the health and physical capabilities of the old (62).

From our point of view, housing as one of the key symbols of the SES for the elderly is 2-fold. On the one hand, given that in China women retire at 55 and men at 60 according to the retirement regulations, and it becomes not appropriate to use occupation or income to explore the SES effects on elders' health. On the other hand, because of filial piety in China that the young generation should support the old generation, to return the care their parents have given them (63), the elderly generally have the “back-feeding” from their children, and this kind of “back-feeding” includes marital and monetary support. Thus, an old person's social class depends on his/her family to some degree, especially with respect to housing conditions. To the best of our knowledge, few studies explicitly explore the potential role of housing conditions in explaining these competing hypotheses of the dynamic patterns of SES differentials in health with age. Therefore, we used housing as the second indicator for SES in our study (64).

In addition, investigating how the educational attainment and housing together have shaped elders' health can help understand how the inconsistency of education and housing condition impacts people's health. According to the status inconsistency theory, individuals whose SES are inconsistent, that is, ranked higher or lower on one dimension than the other dimensions could feel more frustrated and discontented than people whose statuses are consistent on all dimensions (65). For some Chinese elders who are identified as low regarding educational attainment but are ranked high in other dimensions, such as on housing, they could experience more mental health issues and other consequential physical health issues. Although it can be inferred that the effects of SES indicators on different health measures might not be significantly different, effects could be inconsistent due to the status inconsistency. Although an individual's education is likely to remain the same after having accomplished tertiary education (66), housing conditions could significantly change as life unfolds. Thus, due to the similarities and differences between educational attainment and housing condition, how education and housing affect health, especially in old age is still puzzling.

In brief, we investigated three questions: (1) Does the health gradient converge or diverge in old age? (2) Do SES indicators (education and housing) differentiate on health? and (3) Do the effects of SES on elders' health change over time?

## Materials and Methods

### Data Sources

To explore the effects of educational attainment and housing condition on the elderly' health, we used data from China's Inter-census Survey (CIS), a nationally representative survey covering the mainland. In this study, the CIS 2010 sampled 1‰ of the sixth population census in the same logic; the CIS 2015 was also the 1‰ of the population census in 2015, both systematically sampled as the 1‰ of the total population in China. The most outstanding advantage of using CIS data sets is its national representativeness. To explore the heterogeneity of the old, this study narrowed down the sample to elders aged 60 and above.

### Measures

Health. It is measured as self-rated health, which was surveyed between 2010 and 2015. Self-rated health is one of the most frequently used and most popular indicators of health (45). Studies have proved that it is inclusive and accurate in measuring health and other risk factors. Respondents aged 60 and above in the surveys of CIS 2010 and CIS 2015 were asked to rate their health according to a four-item Likert scale, “very healthy,” “healthy,” “unhealthy,” and “very unhealthy”. Since self-rated health was regarded as a general indicator (67–69), we constructed a dummy variable with “very healthy” and “healthy” coded 1, and 0 the otherwise according to previous studies (70, 71).

Socioeconomic status. It is measured by educational attainment and housing condition. Specifically, educational attainment is operationalized as the years of the highest achieved educational level, which we reckon can better capture educational attainment. Because the quantity of education is not equal to the credential (50). The CIS enquired the level of schooling attained by all the respondents and if these respondents fully accomplished their schooling. Educational attainment is captured as the years of schooling only when the respondents have fully accomplished the schooling. Afterward, this value will be modified if he/she is a dropout, according to the human capital theory (72). For example, if an individual has studied in a University but dropped out before graduation, the schooling year of University education was halved, but the schooling year before University remained constant. In order to compare coefficients, it is desirable to standardize educational attainment (in years) as a continuous variable, ranging from *x* to *y*. Regarding the housing condition, it is constructed as an aggregated score based on five aspects, including the housing area, the homeownership (self-owned house or rented house), the building time (inversed assignment), the housing type (one-story house or multi-story house), and hardware facilities indoor (including the kitchen and the toilet) (52, 73). Based on these, housing condition is standardized as a continuous variable.

Control variables: Covariates included in this study are age, survey year, gender, marital status, and province. In this paper, age is measured as a continuous variable. The survey year was a dummy variable, with 2015 coded as 1 and 2010 as 0. Gender was a dummy with female coded as 1 and male as 0. Marital status is coded as a four-categorical variable: married or re-married, never married, divorced, and widowed. Considering the significant socioeconomic disparities among regions in China (26), the province is included as a set of dummy variables.

The sample size was 162,077 in the 2010 data and 205,761 in 2015 data, after deleting the cases, which were missing on the dependent variable and/or key independent variables. The descriptive analysis of variables was shown in Table 1. It is noticeable that the number of elderly people has increased at all ages rapidly, which also illustrates the challenge of aging in China.

**Table 1:** Sample characteristics.

	**Total sample (*****n*** **= 367,838)**		**2010 (*****n*** **= 162,077)**		**2015 (*****n*** **= 205,761)**	
	**Observations**	**Mean/%**	**Observations**	**Mean/%**	**Observations**	**Mean/%**
**Health**						
Very healthy	157,148	42.7	74,917	46.2	82,231	40.0
Healthy	150,716	41.0	62,602	38.6	88,114	42.8
Unhealthy	50,353	13.7	19,842	12.3	30,511	14.8
Very unhealthy	9,621	2.6	4,716	2.9	4,905	2.4
**Age (continuous)**						
60+	367,838	69.4	162,077	69.7	205,761	69.1
**Age (category)**						
60–64	125,762	34.2	52,711	32.5	73,051	35.5
65–69	87,578	23.8	36,521	22.5	51,057	24.8
70–74	63,064	17.1	29,763	18.4	33,301	16.2
75–79	46,973	12.8	22,757	14.1	24,216	11.8
80+	44,461	12.1	20,325	12.5	24,136	11.7
**Educational attainment**	367,838	0.0	162,077	0.0	205,761	0.0
**Housing condition**	366,074	0.0	161,937	0.0	204,137	0.0
**Gender**						
Male	188,909	51.4	83,461	51.5	105,448	51.3
Female	178,929	48.6	78,616	48.5	100,313	48.7
**Marital status**						
Never married	4,896	1.3	2,483	1.5	2,413	1.2
Married or re–married	270,666	73.6	116,710	72.0	153,956	74.8
Divorced	3,423	0.9	1,412	0.9	2,011	1.0
Widowed	88,853	24.2	41,472	25.6	47,381	23.0

### Analytical Strategy

In this study, we first described the health heterogeneity within the elderly group by using two survey years of CIS (2010 and 2015) in order to sketch the overall health differences according to the age groups and the changes from 2010 to 2015. After that, we investigated the associations between two SES measurements (educational attainment and housing condition) and self-rated health. In addition, we explored whether these correlations persisted after half a decade to explore the temporal effects. Furthermore, we evaluated whether SES health difference converges or diverges by age in the late life stage. Finally, we compared the health effects between the two SES indicators to assess whether such variations were statistically significant. Since the dependent variable was binary, our research was based on logistic regressions.

## Results

To describe the heterogeneity of the elderly's health and its tendency in China, we provided the percentage distribution of self-rated health by age as shown in Table 2.

**Table 2:** Percentage distribution of self–rated health by age and survey years.

		**Very healthy**	**Healthy**	**Unhealthy**	**Very unhealthy**
**2010**	60+	46.2	38.6	12.3	2.9
	60–64	63.8	30.2	5.2	0.8
	65–69	52.0	37.7	8.7	1.6
	70–74	38.3	45.2	14.0	2.5
	75–79	29.7	47.0	19.2	4.1
	80+	20.3	43.2	26.5	10.0
**2015**	60+	40.0	42.8	14.8	2.4
	60–64	55.1	37.7	6.5	0.7
	65–69	43.1	44.4	11.3	1.2
	70–74	31.6	48.0	18.4	2.0
	75–79	23.6	47.9	24.8	3.7
	80+	15.4	42.8	32.6	9.2

As shown in Table 2, the proportion of elders who self-rated themselves as “very healthy” decreased rapidly with age, whereas the percentage of “basically good” first rose and then slightly decreased. Regarding the proportions of elders who rated their health status as “unhealthy,” it kept increasing with age. We observed that in 2010, 46.2% of the respondents rated themselves as very healthy, 38.6% as healthy, 12.3% as unhealthy, and only 2.9% as “very unhealthy,” whereas as in 2015, the percentages of the four items were 40.0, 42.8, 14.8, and 2.4%, respectively. Table 2 illustrates that the proportion of elders who reported their health as “unhealthy”; nevertheless, the proportion of elders who chose “healthy” or “unhealthy” was smaller in 2015 than that in 2010. In sum, age is the key factor to influence the health status in old age, and there seem to be some differences between 2010 and 2015. One of the most puzzling observations in our study lies in the increase of those who rated themselves as “unhealthy,” which is discussed in the following analysis.

Furthermore, we explored whether educational attainment and housing condition were associated with health in old age and whether the associations become weaker or stronger over time. In Table 3, Model 1 displayed the estimates of educational attainment on health, with control variables included. Model 2 represented the results of how housing conditions affected health. Model 3 provided the estimates of both educational attainment and housing condition along with their interactions with the survey year.

**Table 3:** Binary logistic regression models of the SES effects.

	**Health**								
	**Model 1**			**Model 2**			**Model 3**		
	**coef**.	**S.E**.	***p*-value**	**coef**.	**S.E**.	***p*-value**	**coef**.	**S.E**.	***p*-value**
Age	−0.079	0.001	0.000	−0.090	0.001	0.000	−0.079	0.001	0.000
Survey year (reference group: 2010)	−0.148	0.011	0.000	−0.156	0.011	0.000	−0.150	0.011	0.000
Educational attainment	0.373	0.008	0.000				0.360	0.008	0.000
Housing condition				0.211	0.006	0.000	0.170	0.009	0.000
Survey year × Educational attainment							0.170	0.011	0.000
Survey year × Housing condition							−0.075	0.011	0.000
Gender (reference group: male)	−0.073	0.010	0.000	−0.146	0.010	0.000	−0.064	0.010	0.000
**Marital status (reference group: Married or re–married)**									
Never married	−0.936	0.034	0.000	−1.159	0.034	0.000	−0.875	0.035	0.000
Divorced	−0.327	0.051	0.000	−0.249	0.050	0.000	−0.287	0.051	0.000
Widowed	−0.321	0.011	0.000	−0.425	0.011	0.000	−0.315	0.011	0.000
Province	control			control			control		
Constant	1.924	0.033	0.000	2.238	0.032	0.000	1.949	0.033	0.000
Observations	367,838			366,074			366,074		

From Table 3, we found that the odds ratio (OR) of well-educated elders who reported themselves as “health” was about 45.2% [OR = exp(0.373) = 1.452], significantly higher than that of the less-educated ones. Similarly, elders who have an increase of the overall housing condition score for every one unit reported about 23.5% [exp(0.211) = 1.235] higher of the OR of being healthy. In other words, the socioeconomically disadvantaged Chinese population is more likely to be unhealthy in their old age. We incorporated both the SES indicators and their interactions with survey year in the full model (Model 3), the log(odds) of the SES remained stable. With respect to two interactions, the survey year interacts with the educational attainment (coef. = 0.170, *p* < 0.000) and survey year interacts with housing condition (coef. = −0.075, *p* < 0.000), both the effects were statistically significant, whereas the directions opposing with each other. For the effect of educational attainment on health, its positiveness waxed over the years, whereas the impact from housing on health waned over time. Therefore, we conclude that the educational attainment and housing condition are important indicators of the self-rated health of the elderly over 60 years in China, and the effect size and direction of these two indicators differentiate over time. In this regard, this study has provided some evidence supporting the association between educational attainment and housing condition and health in old age.

In addition, the control variables also displayed some significance associated with health. Married elders (including married and re-married) were significantly healthier than the other groups. This could be explained by the important role played by the spouse as the caregiver and accompany provider, which is essential to the health in old age (74). The most unexpected finding in our research was that elders in 2010 reported better health status than those in 2015. But this result was similar to the finding of Zeng et al. (2017) that there was a significant increase in the proportion of the Chinese elders aged above 80 who were incapable of physical performance and were generally losing their cognitive capacity (75). The trend toward aging and health probably emerges in China, namely “longer life but worsening health,” which perplexed some developed countries in the late 1970s and early 1980s (76). Zeng et al. (2017) explained that thanks to the socioeconomic development and medical and health advancements over the last several decades, the life expectancy in China has been significantly increased, the process of an individual losing the ability to perform daily self-care activities because of aging has been systematically postponed compared to the older generations. Meanwhile, elders can also live longer with functional limitations and disabilities nowadays (77). This was also supported by the evidence found in our study based on the growing proportion of those who reported “unhealthy” (as shown in Table 2).

Furthermore, using logistic regressions, we investigated whether the SES effects on self-rated health are divergent or convergent among the elders in China. Since the pattern of health inequalities in old age may differ by year, we also ran regression models separately by year, as shown in Table 4. For Model 4, we used data from both of the surveys; in Model 5, we only used the data of 2010; in Model 6, we regressed on only 2015 data. In each model, covariates were included.

**Table 4:** Binary logistic regression models with age pattern.

	**Model 4 (Total sample)**			**Model 5 (2010 sample)**			**Model 6 (2015 sample)**		
	**coef**.	**S.E**.	***p*-value**	**coef**.	**S.E**.	***p*-value**	**coef**.	**S.E**.	***p*-value**
Age	−0.071	0.001	0.000	−0.072	0.002	0.000	−0.070	0.001	0.000
Educational attainment	0.481	0.006	0.000	0.385	0.010	0.000	0.559	0.009	0.000
Age × Educational attainment	−0.002	0.000	0.000	−0.002	0.000	0.000	−0.002	0.000	0.000
Housing condition	0.132	0.006	0.000	0.188	0.010	0.000	0.103	0.007	0.000
Age × Housing condition	−0.003	0.001	0.000	−0.002	0.001	0.112	−0.004	0.001	0.000
Gender (reference group: male)	−0.063	0.010	0.000	−0.021	0.016	0.188	−0.091	0.014	0.000
Never married	−0.855	0.035	0.000	−0.784	0.050	0.000	−0.931	0.049	0.000
Divorced	−0.288	0.051	0.000	−0.392	0.078	0.000	−0.221	0.068	0.001
Widowed	−0.319	0.011	0.000	−0.266	0.017	0.000	−0.362	0.015	0.000
Survey year (reference group: 2010)	−0.168	0.011	0.000	–	–	–	–	–	–
Province	control			control			control		
Constant	1.948	0.033	0.000	1.669	0.045	0.000	2.050	0.047	0.000
Observations	366,074			161,937			204,137		

As shown in Model 4, the coefficient of education attainment was significantly positive (coef. = 0.481, *p* < 0.000), whereas the coefficient of its interaction item with age was significantly negative (coef. = −0.002, *p* < 0.000). That is, the health effects of education attainment became weaker with aging. Therefore, we cannot reject the hypothesis of the convergent effect of educational attainment. Likewise, the effect of housing conditions also decreased with aging because the main effect of housing conditions was significantly positive (coef. = 0.132, *p* < 0.000), whereas the interaction with age was significantly negative (coef. = −0.003, *p* < 0.000). In addition, we found that in the sample of 2010, the interaction item of educational attainment and age was still significantly negative (coef. = −0.002, *p* < 0.000), whereas the effect from housing condition was negative albeit insignificant (coef. = −0.002, *p* > 0.1). By contrast, we found both the interaction items, education attainment with age (coef. = −0.002, *p* < 0.000) and housing condition with age (coef. = −0.004, *p* < 0.000), were significantly negative. In other words, the effect of educational attainment on self-rated health would be always convergent in old age, but the effect of housing conditions would be convergent later. Therefore, we found the pattern of age-as-leveler in old age in our study. And from Table 4, we found that the effects of educational attainment and housing condition may not remain constant, and instead, they varied with age. This helps explain how aging contributes to health inequality.

**Figure 1:**
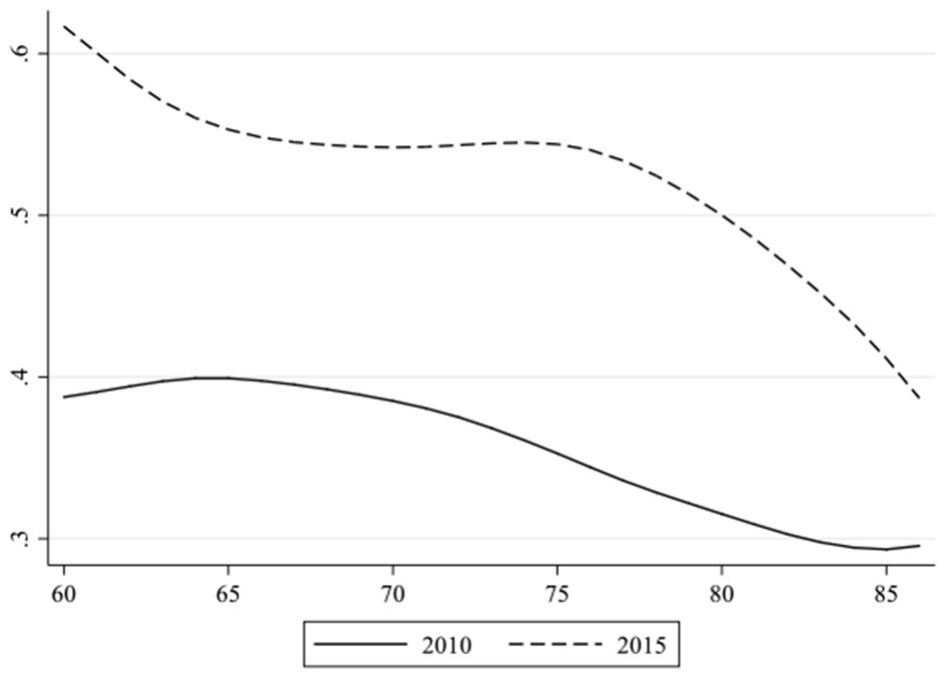
Coefficients of educational attainment on self-rated health at every age by survey year.

**Figure 2:**
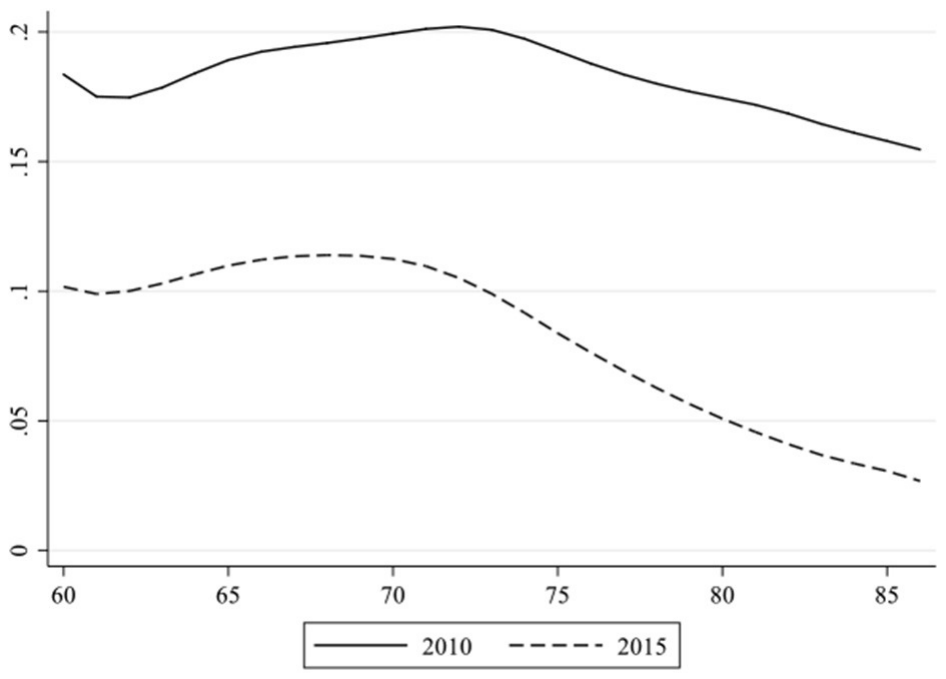
Coefficients of housing condition on self-rated health at every age by survey year.

What are the effects of educational attainment and housing condition on health and how do these two SES indicators change their influence on health over time and age? To answer these questions, we obtained 27 coefficients derived from the logistic regressions at each age point (86 years old and above as the ending point) and plotted out the change of the coefficients of educational attainment separately by age and by survey year, as displayed in Figures 1, 2.

Figure 1 shows that although the two curves slightly fluctuated, the overall tendency decreased both in 2010 and 2015. In other words, with the increase of age, the impact of educational attainment diminished. This is consistent with the convergence effect reported by Herd (39). Furthermore, the curve of 2015 has always been systematically above that of 2010, which also indicated that the impact of education was larger before.

Later, we anatomized Figure 2. These two curves in Figure 2 are similar to those in Figure 1. In both time periods, the coefficients of housing condition diminished with some mild fluctuations between 60 and 73. This is in line with the analysis shown in Table 4 that the convergent effect of housing only appeared in 2015. However, the most outstanding difference in Figure 2 from Figure 1 is that the curve of 2015 is systematically below that of 2010. This indicates that the impact of housing conditions is diminishing.

Several conclusions can be drawn from the above results. First, SES, measured as educational attainment and housing condition, was influential on the self-rated health of Chinese elders. The main differences between educational attainment and housing condition were 2-fold. First, the importance of educational attainment was increasing, whereas it was decreasing for the housing on health; second, the convergence effect of educational attainment and housing condition was becoming increasingly clear over time.

## Discussion

Socioeconomic status has been recognized as the fundamental cause of social disparities and health inequalities (18, 78); however, the disputes of how health inequality was increased by SES over time are accumulating among the life course studies. Although the causal link between SES and health seems to be established, it is noticeable that the role played by the age is still controversial. Some studies suggest that the association between SES and health strengthens with age (divergence effect hypothesis), whereas other studies indicate that it weakens (convergence effect hypothesis). Therefore, in this study, we aimed at figuring out whether the return of SES on health is consistent in old age and whether these effects will increase or decrease in an age-related pattern. Since educational attainment has been widely accepted as a key factor and housing condition has become a new dimension of social stratification, we explored these two SES indicators together in our study. Using data from China's 2010–2015 Inter-census Survey (1‰ sample), which includes a large number of Chinese aged 60, we explored how the effects of educational attainment and housing condition gradients in self-rated health vary by age in China's elderly population and whether they are convergent.

The main findings of this paper were as follows. First, SES was closely associated with the production and reproduction of health throughout the stage of old age. The health of Chinese elders was affected by both the indicators of SES, educational attainment, and housing condition. In the two survey years, access to education and housing both displayed a significant role in regulating the health of Chinese elders. Empirical results in our study suggested that higher SES contributed to better-off health by higher educational attainment and better housing. Second, we found the patterns of age-as-leveler, supporting the convergence hypothesis. The analysis showed that in two survey years, the health effect of educational attainment on the elderly over 60 years old was convergent on age, but the convergence effect of housing appeared later (in 2015). Third, we spotted that educational attainment and housing conditions showed different trends on time. When compared with 2010, the impact of educational attainment increased in 2015, whereas the impact of housing status decreased. This indicates that educational attainment has played an increasingly important role in producing the internal heterogeneity of health among the Chinese elderly. Given the rising scale of the aged population in China, the health of this group will consistently remain an issue, especially in rural China, where the welfare system is still underdeveloped (79–81). Aging for individuals in late life means a rapid decline of individual physical, cognitive, and physiological functions. This would be especially challenging for rural elders. In our study, we also observed the pattern of age-as-leveler of elderly Chinese. The effects of SES varied with age and time, revealing the dynamic association between SES and health.

Although both the educational attainment and housing condition were equally important factors in modifying the self-rated health of the elderly in China, they displayed some differences in terms of how they imposed an impact on health. As we found that from 2010 to 2015, the influence of educational attainment on self-rated health waxed, whereas on housing conditions waned. Due to this, we suggest that the government should increase the accessibility of education for individuals from less-advantaged social backgrounds and provide more affordable housing.

Furthermore, health outcomes differed according to the survey years. This can be explained that the samples in this study are senior Chinese elders, and the average life expectancy has increased tremendously over the past 70 years. Since the rising number of senior elders in China can live longer than the older generations, the local governments in China should improve the healthcare system and medical technology to accommodate the need.

Our research focused on a particular old generation in China. The elders in our research sample represented those who grew up in the most deprived era of China, having suffered from lack of housing, means of subsistence, and healthcare. Due to this, the accumulation of health risks since young may lead to the prevalence of chronic diseases among this old population. Consequently, this may result in the dominating influence of biological factors and the age-as-leveler pattern among this elder cohort. They need more aid and care from society. Regarding whether the gap of education and housing in the different birth cohorts may reproduce or strengthen the health inequality deserves more future studies.

Lastly, this study has some limitations. Because of using the cross-sectional data, we were cautious about establishing the causal link between SES and health. Likewise, the impact of childhood is not included without adequate information. However, our research revealed the convergent effects of SES by age and did not support the cumulative impact of SES on health. Our empirical study can shed some light on how SES reshapes health in old age.

## Data Availability Statement

The original data is credited to National Bureau of Statistics of China. The data analyzed in this study is subject to the following licenses/restrictions: The CIS (1‰ sample) can be requested from Tsinghua China Data Center upon reasonable research request.

## Author Contributions

All authors listed have made a substantial, direct, and intellectual contribution to the work and approved it for publication.

## Conflict of Interest

The authors declare that the research was conducted in the absence of any commercial or financial relationships that could be construed as a potential conflict of interest.

## Publisher's Note

All claims expressed in this article are solely those of the authors and do not necessarily represent those of their affiliated organizations, or those of the publisher, the editors and the reviewers. Any product that may be evaluated in this article, or claim that may be made by its manufacturer, is not guaranteed or endorsed by the publisher.
